# The involvement of krüppel-like transcription factor 2 in megakaryocytic differentiation induction by phorbol 12-myrestrat 13-acetate

**DOI:** 10.1186/s40364-024-00614-9

**Published:** 2024-07-17

**Authors:** Zhen Wang, Zhongwen Liu, Pan Zhou, Xiaona Niu, Zhengdao Sun, Huan He, Zunmin Zhu

**Affiliations:** 1 Department of Hematology, Zhengzhou University People’s Hospital, Henan Provincial People’s Hospital, Zhengzhou, Henan China; 2 Henan University, Kaifeng, Henan China; 3 Zhengzhou University, Zhengzhou, Henan China

**Keywords:** Krüppel-like transcription factor 2, Megakaryocytic differentiation, Phorbol 12-myrestrat 13-acetate, Regulatory mechanism

## Abstract

**Background:**

Megakaryocytic differentiation is a complicated process regulated by a series of transcription factors in a context- and stage-dependent manner. Recent studies have suggested that krüppel-like transcription factor 2 (KLF2) is involved in the control of embryonic erythroid precursor cell differentiation and maturation. However, the function and mechanism of KLF2 in regulating megakaryocytic differentiation remain unclear.

**Methods:**

The expression patterns of krüppel-like transcription factors (KLFs) during megakaryocytic differentiation were identified from public databases. Phorbol 12-myristate 13-acetate (PMA) treatment of the myeloid-erythroid-leukemic cell lines K562 and HEL were used as cellular megakaryocytic differentiation models. A lentiviral transduction system was utilized to achieve the goal of amplifying or reducing KLF2. The expression of KLF2 was examined using real-time PCR and western blot. The impact of KLF2 on the megakaryocytic differentiation of K562 cells was examined by flow cytometry, Giemsa staining, Phalloidin staining and western blot. RNA-sequencing (RNA-seq) and chromatin immunoprecipitation-sequencing (ChIP-seq) technologies were used to identify the KLF2-regulated targets.

**Results:**

KLF2 is increased in the maturation process of megakaryocytes. KLF2 overexpression accelerated the PMA-induced megakaryocytic differentiation, as reflected by an increased percentage of CD41/CD61 cells, an increased number of polyploid cells, and an elevated expression of P21 and P27. KLF2 knockdown exhibited the opposite results, indicating that KLF2 knockdown suppressed the megakaryocytic differentiation. Further, combination of the RNA-seq and ChIP-seq results suggested that chimerin 1 (CHN1) and potassium voltage-gated channel subfamily Q member 5 (KCNQ5) may be target genes regulated of KLF2. Both CHN1 and KCNQ5 knockdown could block the megakaryocytic differentiation to some content.

**Conclusion:**

This study implicated a regulatory role of KLF2 in megakaryocytic differentiation, which may suggest KLF2 as a target for illness with abnormal megakaryocytic differentiation.

**Supplementary Information:**

The online version contains supplementary material available at 10.1186/s40364-024-00614-9.

## Introduction

With an incidence of between 5 and 37%, persistent thrombocytopenia (PT) is one of the most significant side effects of allogenic hematopoietic stem cell transplantation (allo-HSCT) and is closely associated with a patient's poor prognosis [1, 2]. Although the pathophysiology of alloHSCT-PT is not well understood, the majority of patients have aberrant bone marrow megakaryocytes [3]. Megakaryocytes are one of the biggest cells that derive from hematopoietic stem cells (HSCs), making up roughly 0.01% of the nucleated cells in the bone marrow [4]. The production of megakaryocytes is a tightly controlled multi-step process of proliferation and differentiation through a series of transformations, including the common myeloid progenitor cells (CMPs), megakaryocyte-erythroid progenitor cells (MEPs), colony-forming unit (CFU)-megakaryocytes and eventually the mature megakaryocytes [5]. Dysplasia, hypoplasia, and enhanced apoptosis of megakaryocytes or their progenitors are recognized as multifactorial mechanisms underlying PT following allo-HSCT. Theoretical support will come from further understanding of the molecular and cellular mechanisms of megakaryocytic biogenesis.

In the development and polyploidization of megakaryocytes, numerous transcription factors play important roles [6]. The krüppel-like factors (KLFs), a family of zinc finger transcription factors containing highly conserved zinc-finger motifs, have been discovered to be intensely involved in extensive biological processes [7]. The KLFs can act as transcriptional activators [8] or suppressors [9], and some have both aspects to regulate gene expression [10]. Among them, KLF2 has attracted substantial research due to its significance in controlling immune cell activity [11], inflammation [12], proliferation [13], and differentiation [14]. Reports have shown that KLF2 is an important regulator of T cell maturation [15]. KLF2 is involved in the processes of CFU-endothelial cells differentiating into mature endothelial cells [16], and endothelial progenitor cells differentiating into endothelial cells [17]. KLF2 regulates vascular tone and improves the capacity of aged angiogenic cells to generate new vessels [18, 19]. Also, KLF1 and KLF2 collaborate to regulate hematopoietic genes that control the maturation of embryonic erythroid precursor cells [20]. Intriguingly, through two publicly available datasets, we found that KLF2 is expressed in a number of cells throughout the hematopoietic process in healthy individuals, within CFU-megakaryocytes and megakaryocytes having a higher KLF2 expression than in the MEGs. Also, the KLF2 expression is dose-dependently elevated by phorbol 12-myristate 13-acetate (PMA, a megakaryopoiesis inducer) in human myeloid-erythroid-leukemic K562 cells. However, the precise action and mechanism of KLF2 in megakaryopoiesis has not been determined.

In the present study, we examined the KLF2 expression before and after megakaryocytic differentiation induced by PMA in two myeloid-erythroid-leukemic cell lines K562 and HEL, and discovered that KLF2 expression is rapidly elevated after megakaryocytic differentiation. Using a lentiviral transduction system, we then artificially amplified or silenced KLF2 expression in order to investigate its function. Subsequent experiments suggested that KLF2 is required for the differentiation and maturation of megakaryocytes. Further, we used omics analysis to explore the downstream molecules regulated by KLF2. Our findings identify KLF2 as a key regulator of megakaryopoiesis, expanding our understanding of the molecular mechanisms in megakaryocytic differentiation and yielding possible new ideas for the intervention in thrombocytopenia.

## Materials and methods

### Database screening

The expression profiles of KLFs in normal human hematopoiesis (DMAP) were analyzed using data obtained from Bloodspot (https://servers.binf.ku.dk/bloodspot/) [21]. The expression profiles of KLFs in the differentiated megakaryocytes induced by PMA were analyzed using data from the GSE63888 dataset in the Gene Expression Omnibus database (https://www.ncbi.nlm.nih.gov/geo/).

### Cell culture and treatment

Two human myeloid-erythroid-leukemic cell lines, K562 and HEL, were purchased from iCell Bioscience Inc. (Shanghai, China). K562 Cells were cultured in Iscove’s Modified Dulbecco Medium (IMDM, Servicebio Technology Co., Ltd., Wuhan, China) containing 10% fetal bovine serum (FBS, Zhejiang Tianhang Biotechnology Co.,Ltd., Huzhou, China) at 37 °C in a humidified incubator with 95% air and 5% CO_2_, and HEL cells were cultured in Roswell Park Memorial Institute (RPMI)-1640 medium (Solarbio Science & Technology Co., Ltd., Beijing, China) containing 10% FBS at 37 °C in a humidified incubator with 95% air and 5% CO_2_. Cells were validated by short tandam repeats sequencing. Mycoplasma test was performed every three months. To induce megakaryocytic differentiation, 25 nM of PMA was added, and the cells were cultured for 0, 2, 4, and 6 days, respectively.

### Megakaryocytic differentiation detection by flow cytometry

Cells were treated with or without 25 nM of PMA for 0, 2, 4, and 6 days. Then the cells were collected, washed with phosphate balanced solution (PBS), and stained with PE Anti-Human CD41 (Thermo Fisher Scientific Inc., Pittsburgh, PA, USA) and FITC Anti-Human CD61 (Proteintech Group, Inc., Rosemont, IL, USA). Gates were established based on single positive staining, disregarding cell debris in the background. The CD41^+^CD61^+^ cells were then counted. Each experiment was performed in triplicate. Flow cytometry and data interpretation were carried out using NovoCyte from Agilent Technologies Inc. (Santa Clara, CA, USA).

### Real-time PCR

Total RNAs were extracted from cells using TRIpure lysis buffer (BioTeke Corporation, Beijing, China). The extracted RNAs were used as the template for the first-strand cDNA synthesis according to the manufacturer’s protocol (Beyotime Institute of Biotechnology Co., Ltd., Shanghai, China). The mRNA expression was quantified by real-time PCR in Exicycler^TM96^ (Bioneer Corporation, Daejeon, Korea). Gene expression was calculated using the 2^−ΔΔCT^ method and normalized to β-actin (a housekeeping reference gene). The primer sequences were as follows: KLF1, 5’-CCTTGCCCTCCATCAGC-3’ (forward) and 5’-TCATCGTCCTCTTCCTCCC-3’ (reverse); KLF2, 5’-TGCGGCAAGACCTACACCAA-3’ (forward) and 5’-GCACAGATGGCACTGGAATGG-3’ (reverse); KLF4, 5’-CCAGAGGAGCCCAAGCCAAAG-3’ (forward) and 5’-TCCACAGCCGTCCCAGTCA-3’ (reverse); KLF13, 5’-TCCCCGCAGAGGAAGCACAAG-3’ (forward) and 5’-GCGTGCTTGGTCAGGTGGTCG-3’ (reverse); chimerin 1 (CHN1), 5’-ACCTGCTCACTGCGAAAC-3’ (forward) and 5’-GTGGGTCCAAAGACGATT-3’ (reverse); potassium voltage-gated channel subfamily Q member 5 (KCNQ5), 5’-GTTTGGCGTAGTTACGCAGC-3’ (forward) and 5’-AGCTTCTGACTGCTTGATGCT-3’ (reverse).

### Western blot

Total proteins were extracted from cells using RIPA lysis buffer (Solarbio). The protein concentrations were determined using a BCA quantitative kit. The total proteins were separated by sodium dodecyl sulfate–polyacrylamide gel electrophoresis (SDS-PAGE) and then transferred to polyvinylidene fluoride (PVDF) membranes. After blocking with skimmed milk, the membranes were incubated overnight at 4 °C with primary antibodies against KLF2 (1:500, Thermofisher, Cat#PA5-40,591), p21 (1:1000, ABclonal Technology Inc., Cat#A19094), p27 (1:1000, ABclonal, Cat#A5357), and glyceraldehyde-3-phosphate dehydrogenase (GAPDH, 1:20,000, Proteintech, Cat#60,004–1-Ig). Horseradish peroxidase-conjugated goat-anti-rabbit and goat-anti-mouse IgGs (Solarbio) were diluted to 1:3000 and further incubated with the respective membranes at 37 °C for 1 h. Protein blots were visualized with an enhanced chemiluminescence reaction (Solarbio) in a Tanon5200 chemiluminescence apparatus.

### Construction of lentiviral vectors and lentiviral infection

For KLF2 overexpression, the complete coding sequence of the homologous KLF2 was constructed and ligated to a lentiviral expression vector. An empty vector was utilized as a negative control. For KLF2 silencing, two short hairpin RNAs (shRNAs) targeting KLF2 were synthesized and respectively ligated to a lentiviral expression vector. A negative shRNA sequence was used as a control. To produce lentiviral particles, HEK293 cells were pre-seeded and co-transfected with a lentivirus packaging vector, a transfer plasmid, and the constructed lentiviral expression vector. The transfected cells were cultivated for 48 h to generate lentiviral particles. For cell infection, cells that reached a confluence of 80% were added with lentiviral particles at a multiplicity of infection (MOI) of 30 and further cultured for 72 h.

### May-Grünwald-Giemsa staining

Cells were infected with corresponding lentiviral particles for 72 h, then treated with 25 nM of PMA for 6 days. Cells were then collected, washed with PBS, and mounted on glass slides. The morphological differentiation was assessed using a May-Grünwald-Giemsa staining kit, and the results were observed under an inverted microscope.

### Phalloidin staining

Cells were infected with corresponding lentiviral particles for 72 h, then treated with 25 nM of PMA for 6 days. Cells were then collected, washed, mounted on a glass slide, and fixed with 4% paraformaldehyde for 15 min. After rinsing, the slide was immersed in 0.1% Triton X-100 for 30 min at room temperature, followed by three washes with PBS to remove any residual solution. The cells were then stained with anti-stain^TM488^ fluorescent phalloidin (Solarbio) in the dark, and counterstained with 4',6-diamidino-2-phenylindole (DAPI, Aladdin Regents Co. Ltd., Shanghai, China) to label the nucleus. Finally, a small amount of anti-fluorescent quencher was applied to the slide before sealing it. The staining results were observed and captured using a fluorescence microscope.

### Megakaryocytic ploidy analysis by flow cytometry

Cells were infected with corresponding lentiviral particles for 72 h, then treated with 25 nM of PMA for 10 days. Cells were then collected, washed, and resuspended with 70% ethanol for an overnight period at 4 °C. On the next day, the cells were washed and then stained with 500 µL of PI/RNase A staining buffer (KeyGen Biotech. Co. Ltd., Nanjing, China) in the dark for 30 min at 4 °C. Subsequently, the DNA ploidy was examined using a NovoCyte flow cytometer, and cells with > 8N DNA were counted.

#### RNA-sequencing

K562 cells were infected with corresponding lentiviral particles for 72 h, then treated with 25 nM of PMA for 6 days. Then the RNAs were extracted with TRIpure lysis buffer. Total RNAs in cells were examined using an Agilent 2100 bioanalyzer to analyze RNA integrity numbers and RNA concentrations. The samples passing the quality test were used to establish the library. Briefly, RNAs were enriched with oligo (dT) magnetic beads and then fragmented with Fragmentation Buffer. cDNAs were produced from the fragmented RNAs in the M-MuLV reverse transcriptase system and then purified. After being end-repaired, the cDNAs were added with a poly(A) sequence and then ligated into the adapters. AMPure XP beads were used to select fragments of 370 ~ 420 size. PCR amplification was conducted, and the PCR products were purified again using AMPure XP beads to obtain a library. Then, using the Illumina HiSeqTM platform (Illumina, San Diego, CA, USA) with the PE150 strategy, the built-in libraries were sequenced.

The Illumina NovaSeq 6000 platform produced raw picture data that was then transformed into raw reads using CASAVA base calling. Clean reads were obtained after removing adapters and reads with low quality. The clean reads were mapped against the reference genome using the HISAT2 program (v2.0.5). Fragments per kilobase of exon model per million mapped fragments (FPKM) values were computed using HTseq to calculate gene expression levels. Pearson's correlation analysis was used to ascertain the correlation between the samples. Using the DESeq2 R program, differentially expressed genes (DEGs) were determined. Significant differential expression was defined as having pValue < 0.05 and |log2FoldChange|> 1. By comparing DEG entries to those in the Gene Ontology (GO) and Kyoto Encyclopedia of Genes and Genomics (KEGG) databases, the DEGs were functionally annotated.

#### Chromatin immunoprecipitation (ChIP)-sequencing

K562 cells were infected with corresponding lentiviral particles for 72 h, then treated with 25 nM of PMA for 6 days. Then the cross-linked chromatin complexes were captured from the cells and sonicated into fragments. Precipitation was performed with an anti-KLF2 antibody incubated overnight at 4 °C. The DNA–protein complexes were subjected to western blot analysis to confirm the precipitation of KLF2. Then the DNA was purified, end-repaired, and connected to the adapters to build a sequencing library. The library products with sizes 370–420 were enriched, quantified, and sequenced on the Illumina NovaSeq 6000 platform with the PE150 strategy. Input DNA and ChIP DNA were subjected to ChIP-seq.

The Illumina NovaSeq 6000 platform produced raw picture data, which were then transformed into raw reads using bcl2fastq2. Clean reads were obtained after removing adapters and reads with low quality. The clean reads were mapped against the reference genome using the Bowtie software. Genome-wide peak scanning was carried out to obtain the location and sequence information of the peaks on the genome. Peaks with pValue < 0.01 were kept. Peak annotation was performed with ChIPseeker.

#### RNA interference

Knockdown of CHN1 and KCNQ5 was realized by transfection with small interference RNA (siRNA). siRNA against CHN1, KCNQ5, and control siRNA were synthesized by JTS Scientific Inc. (Wuhan, China) and transfected according to the standard protocol. The siRNA sequences were as follows: CHN1 siRNA, 5'-GAAGGACUAUACCGAGUAUTT-3' and 5'-AUACUCGGUAUAGUCCUUCTT-3'; KCNQ5 siRNA, 5'-AACCUCAUUCAGUGUGUUUTT-3' and 5'-AAACACACUGAAUGAGGUUTT-3'; control siRNA, 5'-UUCUCCGAACGUGUCACGUTT-3' and 5'-ACGUGACACGUUCGGAGAATT-3'.

#### Statistical analysis

To determine the statistical significance between two groups, the student's t-test was used. To determine the significance across several groups, a one-way analysis of variance (ANOVA) was used. P < 0.05, 0.01, and 0.001 were denoted by the symbols *, **, and ***, respectively. The standard deviation (SD) was displayed by error bars. Prism 8 by GraphPad was used to present the data.

## Results

### Changes in expression of KLF family members during megakarytogenesis

**Fig. 1:**
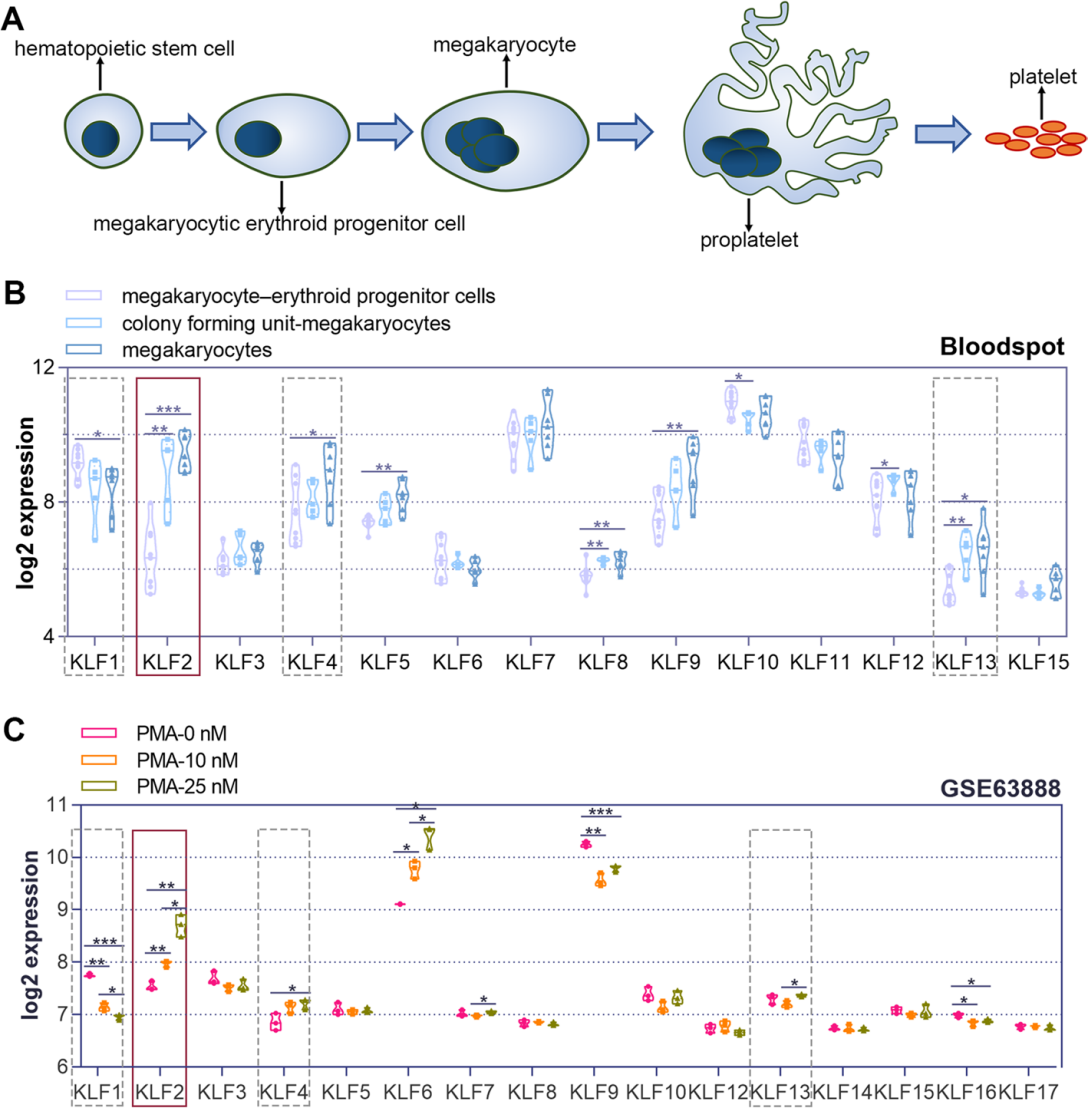
The expression pattern of krüppel like factor (KLF) family members during megakaryopoiesis. **A** Major sequential biological processes characterize megakaryopoiesis and platelet biogenesis. Briefly, hematopoietic stem cells (HSCs) undergo differentiation to produce megakaryocytic erythroid progenitor cells (MEPs). MEPs mature to become polyploid cells after differentiating into immature megakaryocytes. A succession of proplatelet processes with branching ends arises from the demarcation membrane system on mature megakaryocytes. Then, nascent platelets are liberated from the proplatelets, and megakaryocytes go through compartmentalized apoptosis. **B** BloodSpot reflects the expression of krüppel-like factor (KLF) family members in normal human hematopoiesis (DMAP). **C** GSE28703 dataset from the Gene Expression Omnibus (GEO) reflects the expression variation of KLF family members in K562 cells (a human erythroleukemic cell line) after treatment with different concentrations (0 nM, 10 nM, and 25 nM) of phorbol 12-myristate 13-acetate (PMA), an inducer of megakaryopoiesis

In order to illustrate the sequential biological process of megakaryocytic development, we have created a schematic (Fig. 1A). In summary, HSCs differentiate into MEPs, then into immature megakaryocytes, and finally into mature polyploid cells. The demarcation membrane system of mature megakaryocytes generates a series of proplatelets with branching ends, from which new platelets are released. Subsequently, megakaryocytes undergo compartmentalized apoptosis.

Bloodspot is a single-cell transcriptome database of healthy and hematological diseases that covers the gene expression variations of diverse cell states in the hematopoietic system of healthy individuals, including cell states of MEPs, CFU-megakaryocytes and megakaryocytes. We acquired the expression data of members of the KLF family in MEPs, CFU-megakaryocytes, and megakaryocytes. The other components, except for KLF14, KLF16, and KLF17, exhibited varying degrees of relative expression. In mature megakaryocytes, there was an increase in the expression of KLF2, KLF4, KLF5, KLF8, KLF9, KLF12, and KLF13, while there was a decrease in the expression of KLF1 and KLF10 (Fig. 1B). Furthermore, we examined the GSE63888 dataset from the GEO database, which contained information on the RNA-sequencing of PMA-treated K562 cells. In the 25 nM PMA-treated K562 cells, there was a rise in the expression of KLF2, KLF4, KLF6, KLF7, and KLF13, while there was a drop in the expression of KLF1, KLF9, and KLF16 (Fig. 1C). Based on the above results, the expressions of KLF1, KLF2, KLF4, and KLF13 may vary during megakaryocytic differentiation.

### KLF2 is increased in the process of megakaryocytic differentiation induced by PMA

**Fig. 2:**
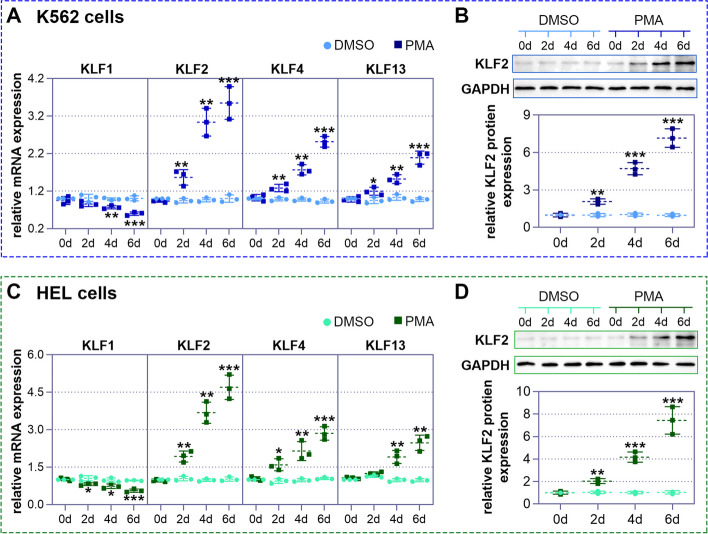
Phorbol 12-myristate 13-acetate (PMA) treatment induces elevated expression of krüppel-like factor 2 (KLF2). Real-time PCR was used to detect the mRNA expression of KLF1, KLF2, KLF4, and KLF13 in two human erythroleukemic cell lines, K562 (**A**) and HEL (**C**), after treatment with 25 nM of PMA for 0 days, 2 days, 4 days, and 6 days, respectively. Among the KLFs, KLF2 shows the most significant change. Subsequently, western blot analysis was used to detect the protein expression of KLF2 in K562 (**B**) and HEL (**D**) cells at these time points, respectively

To determine which KLF is suitable for this study, we examined their expression during megakaryopoiesis experimentally. Two human erythroleukemic cell lines, K562 and HEL, were treated with 25 nM PMA for 0, 2, 4, and 6 days, respectively. Over time, these cells exhibited typical features of megakaryocytes, with a noticeable elevation in the expression of surface markers CD41 (ITGA2B) and CD61 (ITGB3) (Sup. Figure-1). Next, we examined the expression of KLF1, KLF2, KLF4, and KLF13 through real-time PCR. Consistent with the database results, PMA treatment induced KLF2, KLF4, and KLF13 expression, but reduced KLF1 expression in both cell lines (Fig. 2A and C). Among these KLFs, KLF2 showed the most significant change. Subsequently, western blot analysis was performed to validate the KLF2 expression in K562 and HEL cells. As shown in Fig. 2B and D, PMA treatment time-dependently increased the KLF2 protein expression. The potential significance of KLF2 in megakaryocytic differentiation is suggested by differences in KLF2 expression.

### KLF2 influences the formation of megakaryocytes

**Fig. 3:**
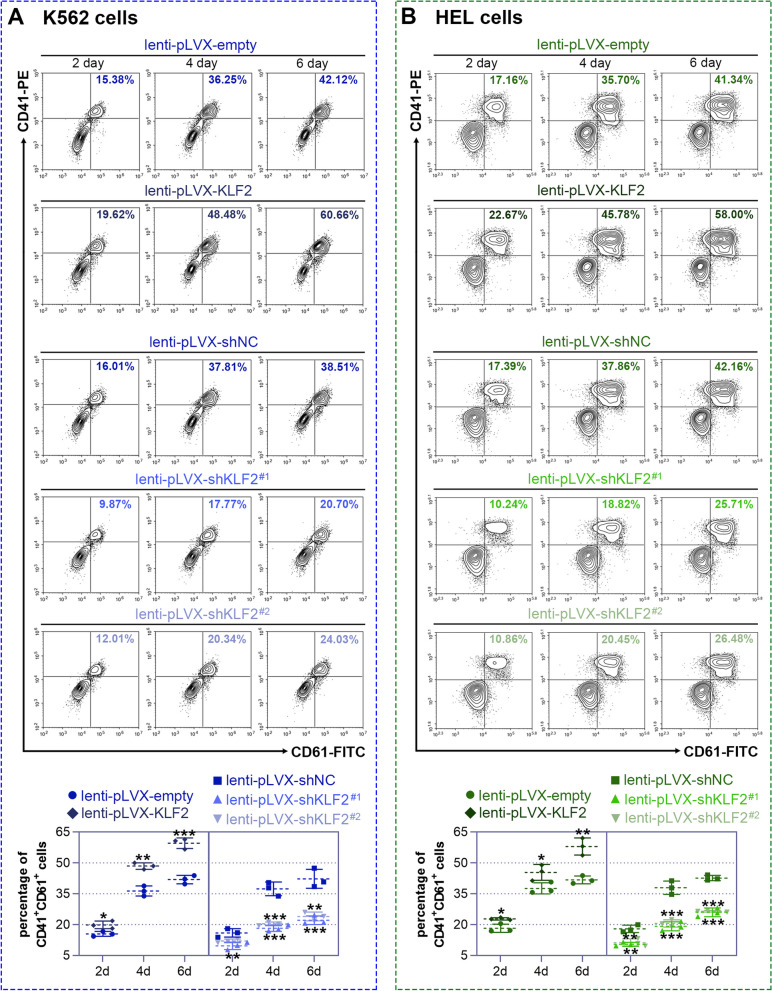
Krüppel-like factor 2 (KLF2) regulates the expression of megakaryocytic markers CD61 and CD41. The lentiviral vectors harboring pLVX-IRES-Puro encoding KLF2 (lenti-pLVX-KLF2), two pLVX-shRNAs against KLF2 (lenti-pLVX-shKLF2^#1^/^#2^), and their control vectors were respectively constructed. The lentiviral particles were infected into K562 or HEL cells for 72 h. Subsequently, the cells were treated with 25 nM phorbol 12-myristate 13-acetate (PMA) and further incubated for 2, 4, and 6 days. Representative contour plots were used to identify CD41^+^/CD61^+^ cells in K562 (**A**) cells and HEL (**B**) cells

**Fig. 4:**
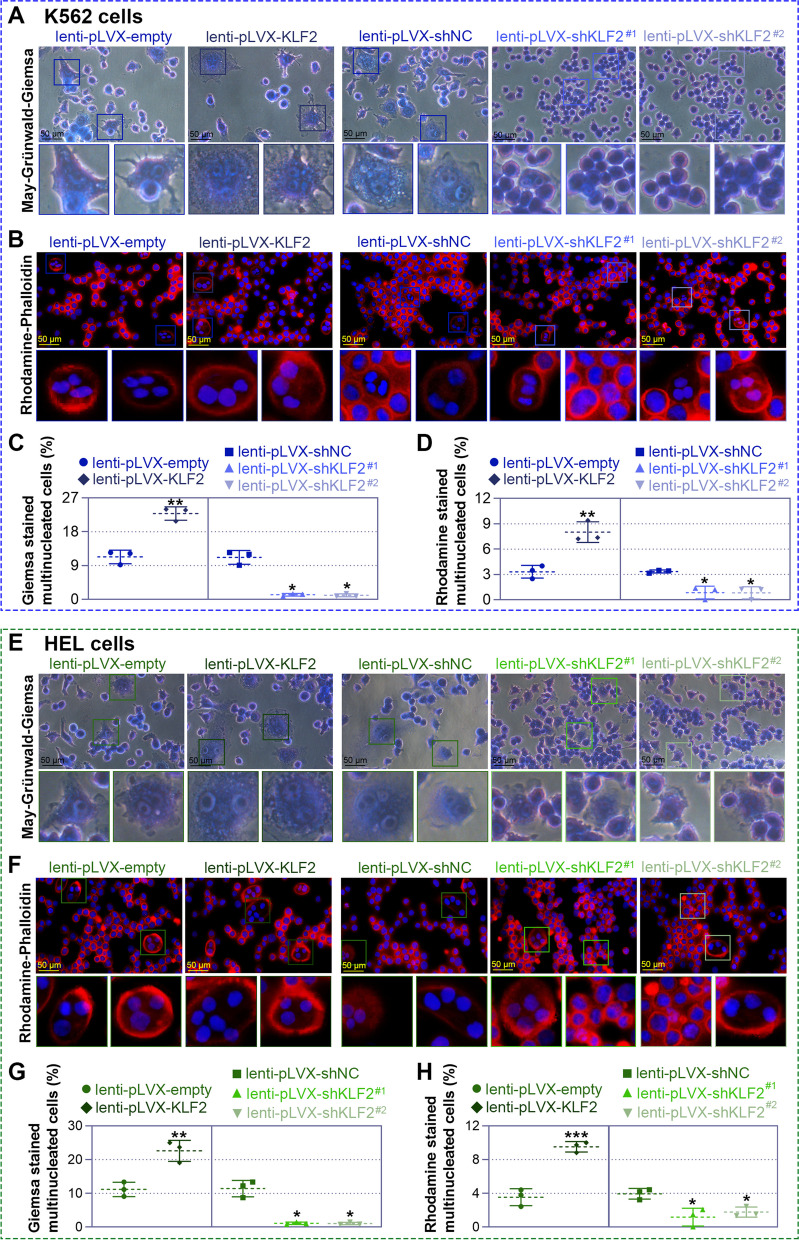
Krüppel-like factor 2 (KLF2) regulates the formation of megakaryocytes. Lentiviral particles were infected into K562 or HEL cells for 72 h, followed by treatment with 25 nM phorbol 12-myristate 13-acetate (PMA) for an additional 6 days to induce megakaryocytic differentiation. The differentiation was assessed using May-Grünwald Giemsa staining (400 × original magnification) to visualize the morphology of K562 (**A**) and HEL (**E**) cells. Two enlarged views are presented below. The number of multinucleated cells stained by Giemsa was quantified (**C** and **G**). Representative immunofluorescence microscopy images (400 × original magnification) of the differentiated megakaryocytes in K562 (**B**) and HEL (**F**) cells are shown. The cells were stained with Rhodamine Phalloidin for F-actin (red) and 4',6-diamidino-2-phenylindole (DAPI) for the nucleus (blue). Two enlarged views are presented below. The number of multinucleated cells stained by Phalloidin was quantified (**D** and **H**)

Next, to investigate the functional role of KLF2 in regulating the production of megakaryocytes, we artificially overexpressed or knocked down KLF2's expression using a lentiviral method. Initially, we assessed the effect of KLF2 on the expression of megakaryocytic surface markers CD41 and CD61 at 2, 4, and 6 days post PMA induction in both K562 and HEL cells. Cells with KLF2 overexpression showed a higher proportion of CD41^+^/CD61^+^ cells, while KLF2 knockdown suppressed the expression of CD41/CD61 (Fig. 3). Subsequently, we employed May-Grünwald-Giemsa staining and phalloidin-Rhodamine staining to visually examine cell morphology and multinucleation. As depicted in Fig. 4, KLF2 overexpression led to the appearance of more multinucleated cells after 6 days post PMA treatment, while this process was significantly impeded by KLF2 knockdown. These results indicate that KLF2 may have a positive impact on megakaryopoiesis.

KLF2 influences DNA ploidy and the expression of related proteins.

**Fig. 5:**
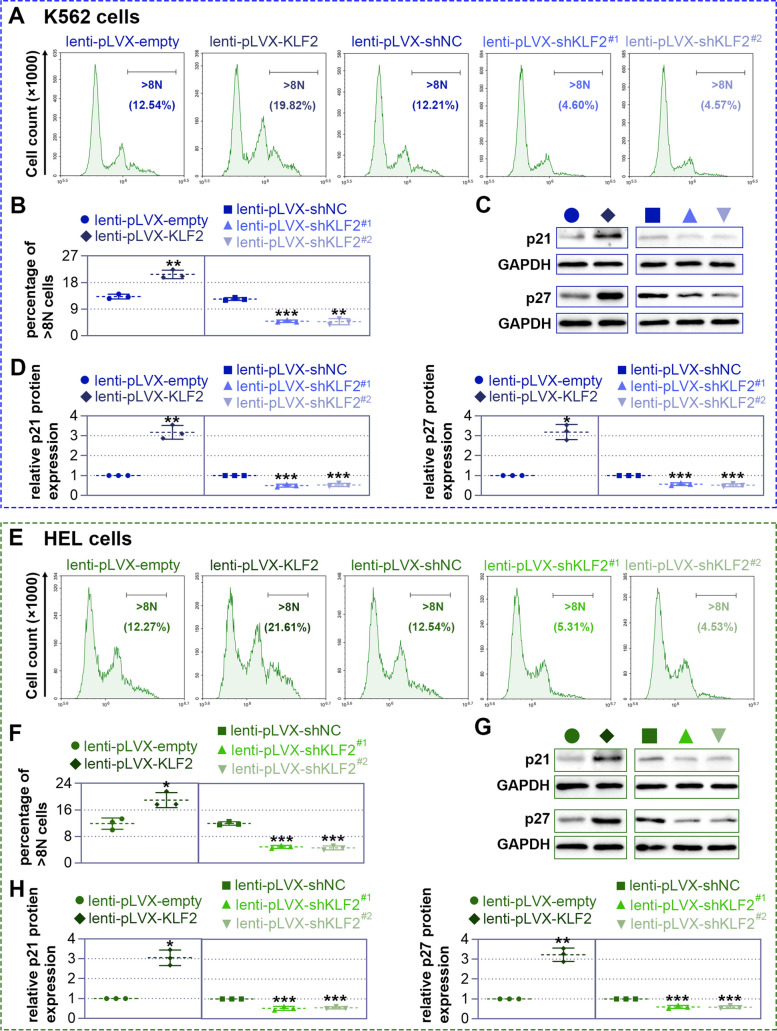
Krüppel-like factor 2 (KLF2) regulates DNA ploidy and the expression of related proteins. Lentiviral particles were infected into K562 or HEL cells for 72 h, and the cells were treated with 25 nM phorbol 12-myristate 13-acetate (PMA) for another 10 days. Subsequently, DNA ploidy was assessed by flow cytometry (**A** and **E**), and cells with > 8N DNA were counted (**B** and **F**). For the detection of megakaryocytic markers p21 and p27, the lentiviral particles were infected for 72 h, and cells were treated with PMA for another 6 days. Western blot analysis was then performed to detect the expression of p21 and p27 in K562 (**C** and **D**) and HEL (**G** and **H**) cells, respectively

Further, we examined DNA ploidy and the expression of related proteins to support the effect of KLF2 on megakaryocytic differentiation. In general, megakaryocytes have DNA content over 8N. Flow cytometry with PI staining showed that KLF2 overexpression significantly increased the number of > 8N cells after 6 days of PMA induction in K562 (Sup. Figure 2) cells, while KLF2 silencing exhibited the opposite results. However, the induction period of PMA seemed a little short because the control groups had only 5% of > 8N cells. Therefore, we treated cells with PMA for 10 days to induce megakaryocytic differentiation again. In this way there were over 10% of > 8N cells. The results showed similar results that KLF2 overexpression markedly increased the > 8N cells, but KLF2 knockdown significantly reduced the > 8N cells in both K562 (Fig. 5A and B) and HEL (Fig. 5E and F) cells.

In order to be consistent with the other PMA treatment period, we still chose to detect p21 and p27 expression after 6 days of PMA treatment. The results showed that KLF2 overexpression promoted, but KLF2 knockdown inhibited the expression of p21 and p27 in K562 (Fig. 5C and D) and HEL (Fig. 5G and H) cells. These findings together demonstrate that KLF2 influences megakaryocytic differentiation.

### KLF2 leads to large transcriptome changes in the differentiated megakaryocytes.

Subsequently, we used RNA-seq on K562 cells that were infected with KLF2-overexpressing lentiviruses or empty lentiviruses to further investigate downstream molecules that KLF2 may affect. Each group contained five biological replicates. In this study, gene expression was calculated using FPKM values. We discovered that the relative densities of the majority of genes were typically similar throughout the samples by comparing the density distribution curves (Sup. Figure 3A) and Pearson correlation analysis (Sup. Figure 3B) of the samples. The principal component analysis (PCA) plot showed that samples from the same group were grouped together (Sup. Figure 3C), indicating the accuracy of the data.

**Fig. 6:**
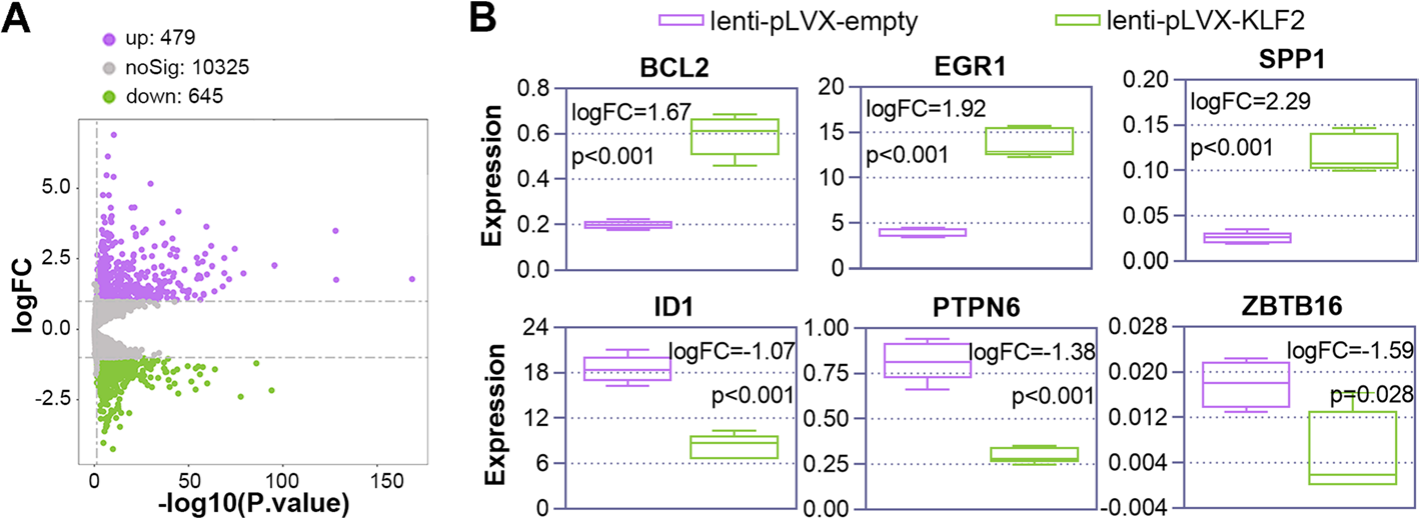
krüppel like factor 2 (KLF2) leads to significant transcriptome changes and alterations of megakaryocytic differentiation-related factors. K562 cells were infected with lenti-pLVX-empty and lenti-pLVX-KLF2 for 72 h, then treated with 25 nM PMA for 6 days to induce megakaryopoiesis. mRNA-sequencing was performed to explore the changed transcriptome expression patterns induced by KLF2 overexpression. **A** A volcano map of the differentially expressed genes (DEGs) between the lenti-pLVX-empty and lenti-pLVX-KLF2 groups. DEGs were screened based on a *p*-value < 0.05 and |log2 Fold Change|> 1. Purple dots represent the up-regulated DEGs, and green dots represent the down-regulated DEGs in the lenti-pLVX-KLF2 group. **B** The expression of megakaryocytic differentiation-related factors, including BCL2, EGR1, SPP1, ID1, PTPN6, and ZBTB16, are presented by box diagrams

Then, we set the criteria of pValue < 0.05 and |log2FoldChange|> 1 to screen the DEGs. A total of 1,124 DEGs were retrieved (479 upregulated and 645 downregulated), and these genes were displayed in a volcano map (Fig. 6A). Among them, we selected several genes that have been reported to regulate megakaryocytic differentiation, including the up-regulated genes B-cell lymphoma 2 (BCL2), early growth response 1 (EGR1), and secreted phosphoprotein 1 (SPP1), as well as the down-regulated genes inhibitor of DNA binding 1 (ID1), protein tyrosine phosphatase non-receptor type 6 (PTPN6), zinc finger and BTB domain containing 16 (ZBTB16). The expression of these genes was displayed in Fig. 6B.

Following that, GO and KEGG annotations were obtained to analyze the functional distribution characteristics of the upregulated or downregulated DEGs. A GSEA analysis was also conducted based on GO and KEGG. Certain annotations, such as cell cycle and DNA replication, may play a role in the regulation of KLF2 in megakaryopoiesis (Sup. Figure 4).

### Identification of KLF2 transcriptional targets according to a combination of ChIP-seq and RNA-seq results

**Fig. 7:**
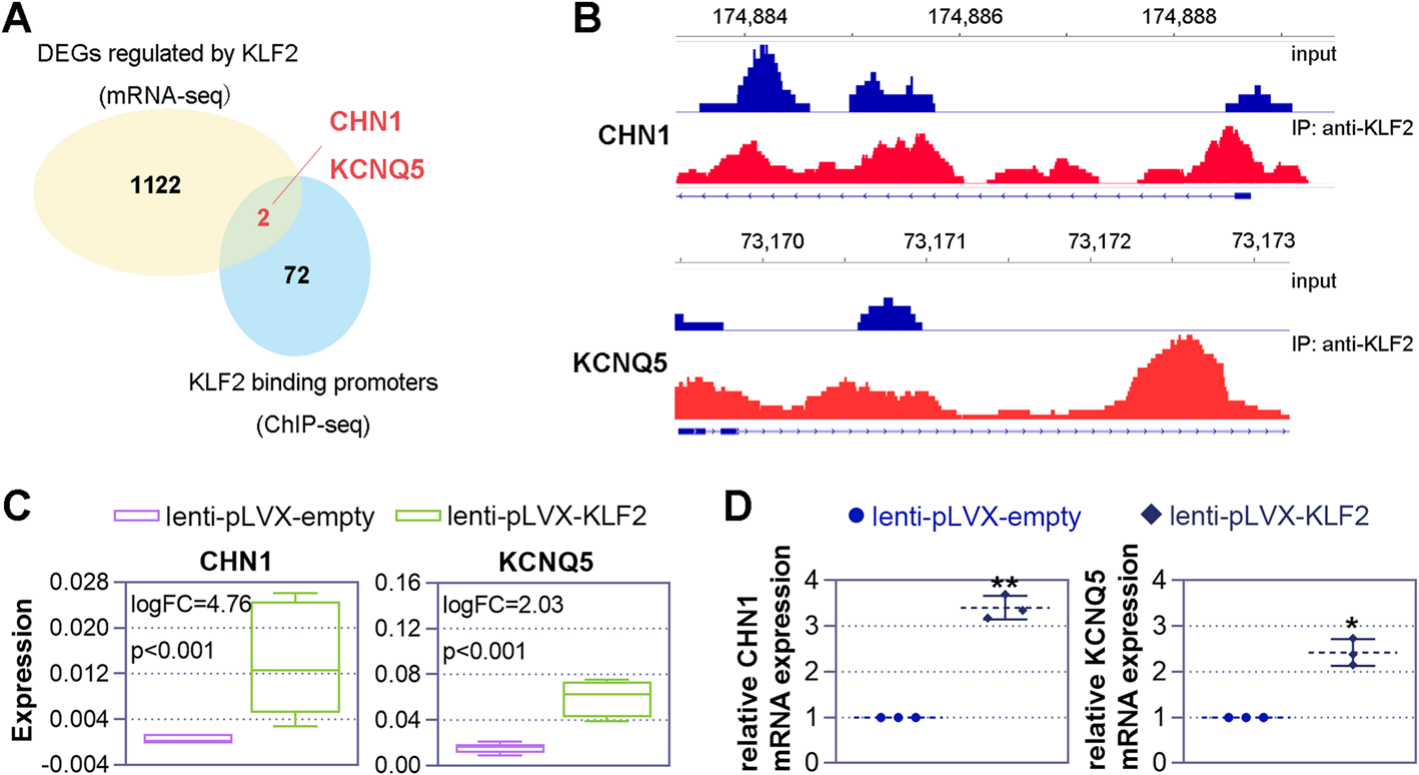
Combining mRNA-sequencing (mRNA-seq) and chromatin immunoprecipitation-sequencing (ChIP-seq) to identify genes regulated by krüppel-like factor 2 (KLF2). **A** A Venn diagram illustrates the overlap of DEGs by mRNA-seq and promoters bound by ChIP-seq. Two genes, chimerin 1 (CHN1) and potassium voltage-gated channel subfamily Q member 5 (KCNQ5), are identified at this intersection. **B** The peaks of CHN1 and KCNQ5 between the anti-KLF2 group and the input group are displayed. **C** Box diagrams present the expression levels of CHN1 and KCNQ5 from mRNA-seq. **D** K562 cells were transduced with lentiviral particles and then treated with 25 nM phorbol 12-myristate 13-acetate (PMA) for 6 days. Subsequently, real-time PCR was performed to detect the expression of CHN1 and KCNQ5

We further sought to find KLF2 transcriptional targets to investigate how KLF2 initiates megakarytogenesis. To achieve this goal, we identified the KLF2 binding sites using ChIP-seq. 182 KLF2 binding peaks, and the distribution of binding sites across the entire chromosome is shown in Sup. Figure 5A. These binding regions are primarily located in the promoter, intron, and distal intergenic regions (Sup. Figure 5B). Since we focus on the promoter region, we present the heatmap and curve of the peak in the promoter region (Sup. Figure 5C). We identified 75 target genes in these regions. Then, by combining the results of mRNA-seq, we generated a Venn diagram to demonstrate the overlap of DEGs by mRNA-seq and promoters bound by ChIP-seq. At this intersection, we identified two genes, CHN1 and KCNQ5 (Fig. 7A). The ChIP-binding peaks of CHN1 and KCNQ5 are displayed in Fig. 7B, and the mRNA-seq expression data of CHN1 and KCNQ5 in empty and KLF2 overexpressed K562 cells is presented in Fig. 7C. Furthermore, we conducted real-time PCR analysis to validate the expression of CHN1 and KCNQ5 after overexpressing KLF2 in K562 cells, 6 days post PMA treatment (Fig. 7D).

### Both CHN1 and KCNQ5 may play a role in megakaryocyte differentiation

**Fig. 8:**
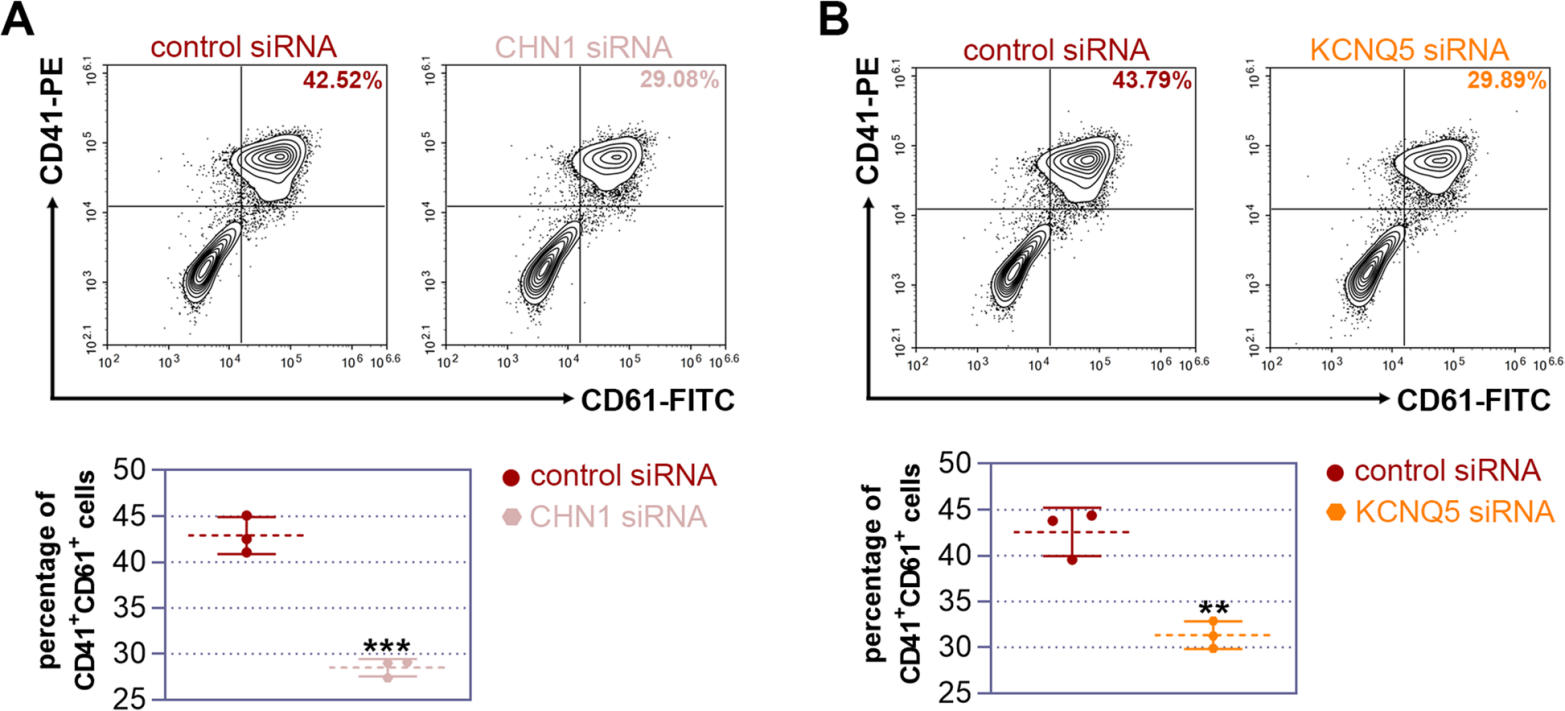
Chimerin 1 (CHN1) and potassium voltage-gated channel subfamily Q member 5 (KCNQ5) may influence megakaryocyte differentiation. siRNAs targeting CHN1 or KCNQ5 were synthesized separately and then transfected into K562 cells along with their respective control siRNAs. Twenty-four hours after transfection, the cells were treated with 25 nM phorbol 12-myristate 13-acetate (PMA) for 6 days. Representative contour plots were used to identify CD41^+^/CD61^+^ cells in the CHN1 siRNA or KCNQ5 siRNA transfected cells, respectively

Intriguingly, the functions of CHN1 and KCNQ5 in the process of megakaryocyte differentiation have been revealed. Therefore, we explored their potential roles in regulating megakaryocyte differentiation. siRNAs targeting CHN1 or KCNQ5 were synthesized separately and then transfected into K562 cells along with their respective control siRNAs. Twenty-four hours after transfection, the cells were treated with 25 nM PMA for 6 days. Then, flow cytometry was performed to detect the expression of CD41 and CD61. The results indicated that both CHN1 and KCNQ5 knockdown by siRNAs resulted in a lower proportion of CD41^+^/CD61^+^ cells (Fig. 8), suggesting that CHN1 and KCNQ5 may play roles in regulating megakaryocyte differentiation.

## Discussion

Gradually, the significance of transcription factors in controlling hematopoietic differentiation has come to light [22–24]. The KLF family, a group of transcription factors reported to play regulatory roles in the proliferation and differentiation of hematopoietic cells, has attracted our attention [25–27]. In the present study, we found that the expression of KLF2, a member of the KLFs, was significantly increased with the maturation of megakaryocytes. Further investigations demonstrated that the differentiation potential of megakaryocytes was promoted by KLF2 overexpression, whereas decreased by KLF2 knockdown. CHN1 and KCNQ5 were identified as two target molecules regulated by KLF2 through RNA-seq and ChIP-seq analyses.

The KLF family has 17 known protein members (KLF1 ~ KLF17), which harbor a high level of conservatism [28]. All the KLFs contain three C2H2 zinc finger structures in the carboxyl end, and each zinc finger structure contains two highly conserved Cys and two His residues [29]. They prefer to bind GC-rich sequences such as "CACCC-box" or "GC-box" and act as transcription factors [30]. Herein, we downloaded human hematopoietic single-cell gene expression profile data from a public database, as well as sequencing data of gene expression in PMA-treated K562 cells, and used bioinformatics methods to evaluate the expression level of the KLFs. We found that the expressions of KLF1, KLF2, KLF4, and KLF13 varied during the maturation of megakaryocytes. Combined with the findings of literature research, we chose KLF2 for the subsequent investigation.

The KLF2 gene, which encodes 354 amino acids, was first cloned by Lingrel and colleagues [31]. Studied have documented that KLF2 is involved in osteoblast differentiation [32], neural differentiation [33], and endothelial colony forming cell differentiation [16]. Notably, in Wang’s paper, KLF2 is reported to be positively involved in the DT-13-induced acute myeloid leukemia cell apoptosis and differentiation [34]. The human leukemia cell lines, K562 and HEL, have been reported to harboring the ability to differentiate into erythrocytes, megakaryocytes and mononuclear macrophage lineage [35, 36]. Therefore, they have been utilized as superior cell models in the discipline of hematopoietic system research. In this paper, using the K562 and HEL cells, we found KLF2 is time-dependently increased after PMA treatment, which is consistent with the results of database screening. Further, we investigated the function of KLF2 in the directional differentiation of megakaryocytes through gene overexpression and knockdown technologies.

In the process of differentiation and development of lineages, the expression of some molecules on the cell surface is constantly changing, and such landmark molecules are relatively specific to different lineages, so the diverse stages of cell differentiation can be judged by examining the markers [37, 38]. For megakaryocytic differentiation, GPIIIa (CD61) first appears in the prokaryotic megakaryocytes, followed by GPIIb/IIIa (CD41), GPIb (CD42b) and other proteins. CD61 is a membrane glycoprotein that binds to CD41 to form the heterodimer complex gpIIb/IIIa (CD41/CD61), which is mainly expressed in platelets and megakaryocytes [39]. These glycoproteins persist, and their expression increases during the differentiation and maturation of megakaryocytes [40]. An increase in CD41/CD61 expression has been regarded as a fate of megakaryocytic differentiation [41]. Herein, we also use these two markers, CD41 and CD61, to distinguish the megakaryocytic differentiation. Intriguingly, we found that KLF2 overexpression increased, but KLF2 knockdown decreased the number of CD41/CD61 cells. In addition, another characteristic of megakaryocytesis the presence of polyploidy, that is, the presence of multiple (usually four or more) nuclei in a single cell [42]. The results of May-Grunwald-Giemsa staining, Rhodamine-Phalloidin staining, and flow cytometry intuitively showed that KLF2 overexpression induced more polyploid cells, whereas KLF2 silencing suppressed cell polyploidy. Furthermore, it is reported that the ectopic expression of p21 or p27 led to induction of megakaryocytic differentiation [43, 44]. We also found that KLF2 overexpression increased the protein expression of p21 and p27, while KLF2 knockdown showed the opposite results. These findings together demonstrate that KLF2 is critical for the megakaryocytic differentiation.

KLF2 works as a transcription factor that binds to the promoters of numerous genes. By controlling whether transcription is turned on or off, KLF2 influences the expression of the genes it interacts with. We searched for the KLF2 targets using high-throughput sequencing technologies including RNA-seq and ChIP-seq to determine which genes are regulated by KLF2. A total of 1,124 DEGs were found after we compared the transcriptome profiles of K562 cells with and without KLF2 overexpression. We found that KLF2 overexpression alters the expression of certain molecules that associated with megakaryocyte differentiation, including the up-regulated DEGs such as BCL2 [45], EGR1 [46], SPP1 [47], and down-regulated DEGs such as ID1 [48], PTPN6 [49], and ZBTB16 [50]. Functional enrichment analysis revealed that the DEGs were connected to a variety of biological pathways and processes. In addition, we discovered 75 target genes by utilizing ChIP-seq to locate the KLF2 binding site. The results of the RNA-seq and ChIP-seq experiments together revealed that KLF2 may have direct targets in CHN1 and KCNQ5. Involved in cytoskeletal control is CHN1, a Ras-related Rho GTPase-activating protein [51], and KCNQ5, a voltage-dependent non-inactivating K-ion channel [52]. Further, we examined the function of these two molecules on megakaryocytic differentiation, and discovered that they may also be potential in regulating megakaryocytic differentiation.

There are several restrictions on this study. First, we solely looked into KLF2's function during the final stage of megakaryocytic differentiation. Uncertainty exists regarding other members’ function and the dynamic process of hematopoietic stem cell differentiation into platelet. Second, we exclusively examined KLF2's expression pattern and functional role in cell lines. These observations warrant further investigation using primary cell models or clinical data. Third, even though we have examined the KLF2 targets using sequencing techniques, more fundamental experimental confirmation of the targets is still needed. However, given that we suggest a novel molecular mechanism of megakaryocytic differentiation, namely that KLF2 could facilitate megakaryocytic differentiation, our results are still intriguing and encouraging. Focusing on KLF2 may become a valuable strategy for treating diseases associated with thrombocytopenia.

## Conclusion

**Fig. 9:**
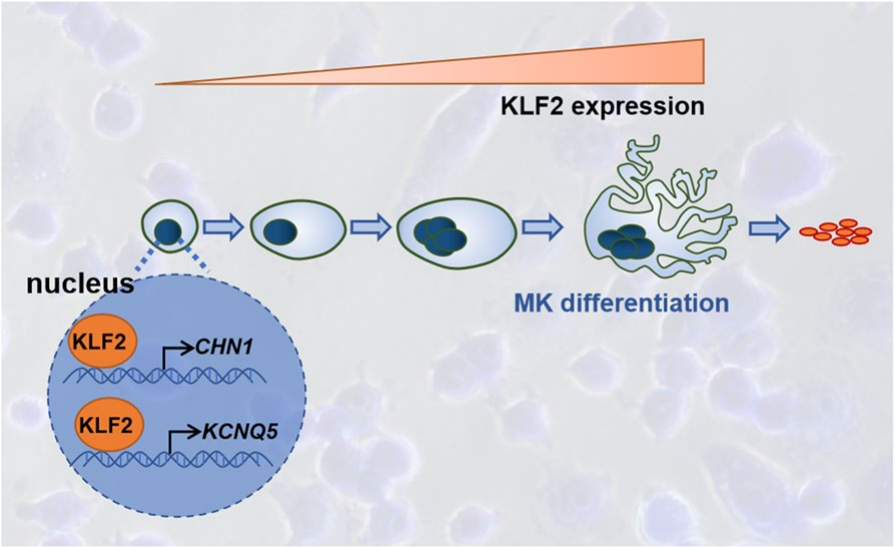
KLF2 is involved in megakaryocyte differentiation through transcriptional activation of CHN1 and KCNQ5

In summary, this study provides evidence that KLF2 is required for the differentiation of megakaryocytes, partly through its direct binding to CHN1 and KCNQ5. The regulatory mechanism diagram is presented in Fig. 9. These findings expand our understanding of the molecular mechanisms in megakaryocytic differentiation and yield possible new ideas for intervention in thrombocytopenia.

### Supplementary Information

Supplementary Material 1. Sup. Figure-1. Phorbol 12-myristate 13-acetate (PMA) treatment induces the expression of the megakaryocytic markers CD61 and CD41. Representative contour plots were used to identify CD41+/CD61+ cells in two human erythroleukemic cell lines, K562 (A) and HEL (B), after treatment with 25 nM of PMA for 2 days, 4 days, and 6 days.

Supplementary Material 2. Sup. Figure-2. Krüppel-like factor 2 (KLF2) regulates DNA ploidy after 6 days of phorbol 12-myristate 13-acetate (PMA) treatment. Lentiviral particles were infected into K562 cells for 72 h, and the cells were treated with 25 nM phorbol 12-myristate 13-acetate (PMA) for another 6 days. Subsequently, DNA ploidy was assessed by flow cytometry (A), and cells with > 8N DNA were counted (B).

Supplementary Material 3. Sup. Figure-3. krüppel-like factor 2 (KLF2) leads to significant transcriptome changes. K562 cells were infected with lenti-pLVX-empty and lenti-pLVX-KLF2 for 72 h, then treated with 25 nM PMA for 6 days to induce megakaryopoiesis. mRNA-sequencing was performed to explore the changed transcriptome expression patterns induced by KLF2 overexpression. A. Overall expressed patterns. The abscissa represents different samples, and the vertical axis represents the centered log2 (FPKM + 1). B. A heatmap of the correlation in the overall gene expression among samples. C. A principal component analysis (PCA) plot based on the gene expression profile among samples. PC1 represents 41.49% variance, and PC2 represents 15.4% variance.

Supplementary Material 4. Sup. Figure-4. Functional enrichment suggests the biological processes and pathways influenced by krüppel-like factor 2 (KLF2). A. Gene Ontology (GO) and Kyoto Encyclopedia of Genes and Genomes (KEGG) enrichment analysis of the up-regulated and down-regulated differentially expressed genes (DEGs). The top 7 enriched pathways are shown in a bar chart. B. Gene Set Enrichment Analysis (GSEA) shows the top 5 enriched GO terms and 3 enriched KEGG pathways.

Supplementary Material 5. Sup. Figure-5. Chromatin immunoprecipitation-sequencing (ChIP-seq) suggests genes that bind with krüppel like factor 2 (KLF2). K562 cells were infected with lenti-pLVX-KLF2 for 72 h, then treated with 25 nM PMA for 6 days to induce megakaryopoiesis. ChIP-seq was performed to explore the binding DNAs of KLF2. A. The location of all ChIP peaks over the chromosome. B. Peaks annotated to the expressed genes are categorized into regions according to the distance from their transcription start site (TSS). A pie chart shows the percentage of peaks in each region. C. Heatmap of the KLF2 ChIP-seq signals at gene promoters (-3 kb ~ 3 kb).

## Data Availability

No datasets were generated or analysed during the current study.

## Abbreviations

allo-HSCT: allogenic hematopoietic stem cell transplantation
ANOVA: analysis of variance
BCL2: B-cell lymphoma 2
CFU: colony-forming unit
ChIP: chromatin immunoprecipitation
CHN1: chimerin 1
CMP: common myeloid progenitor cell
DAPI: 4',6-diamidino-2-phenylindole
DEG: differentially expressed gene
EGR1: early growth response 1
FBS: fetal bovine serum
FPKM: fragments per kilobase of exon model per million mapped fragments
GAPDH: glyceraldehyde-3-phosphate dehydrogenase
GO: Gene Ontology
HSC: hematopoietic stem cell
ID1: inhibitor of DNA binding 1
IMDM: Iscove’s Modified Dulbecco Medium
KCNQ5: potassium voltage-gated channel subfamily Q member 5
KEGG: Kyoto Encyclopedia of Genes and Genomics
KLF: krüppel-like transcription factor
MEP: megakaryocyte-erythroid progenitor cell
MOI: multiplicity of infection
PBS: phosphate balanced solution
PCA: principal component analysis
PMA: phorbol 12-myristate 13-acetate
PT: persistent thrombocytopenia
PTPN6: protein tyrosine phosphatase non-receptor type 6
PVDF: polyvinylidene fluoride
RPMI: Roswell Park Memorial Institute
SD: standard deviation
SDS-PAGE: sodium dodecyl sulfate-polyacrylamide gel electrophoresis
shRNA: short harpin RNA
siRNA: small interference RNA
SPP1: secreted phosphoprotein 1
ZBTB16: zinc finger and BTB domain containing 16
