# Supplementary figures and images for: The involvement of krüppel-like transcription factor 2 in megakaryocytic differentiation induction by phorbol 12-myrestrat 13-acetate
Source: Biomark Res. 2024 Jul 17;12:65. doi: 10.1186/s40364-024-00614-9 (PMC11253501; doi:10.1186/s40364-024-00614-9)

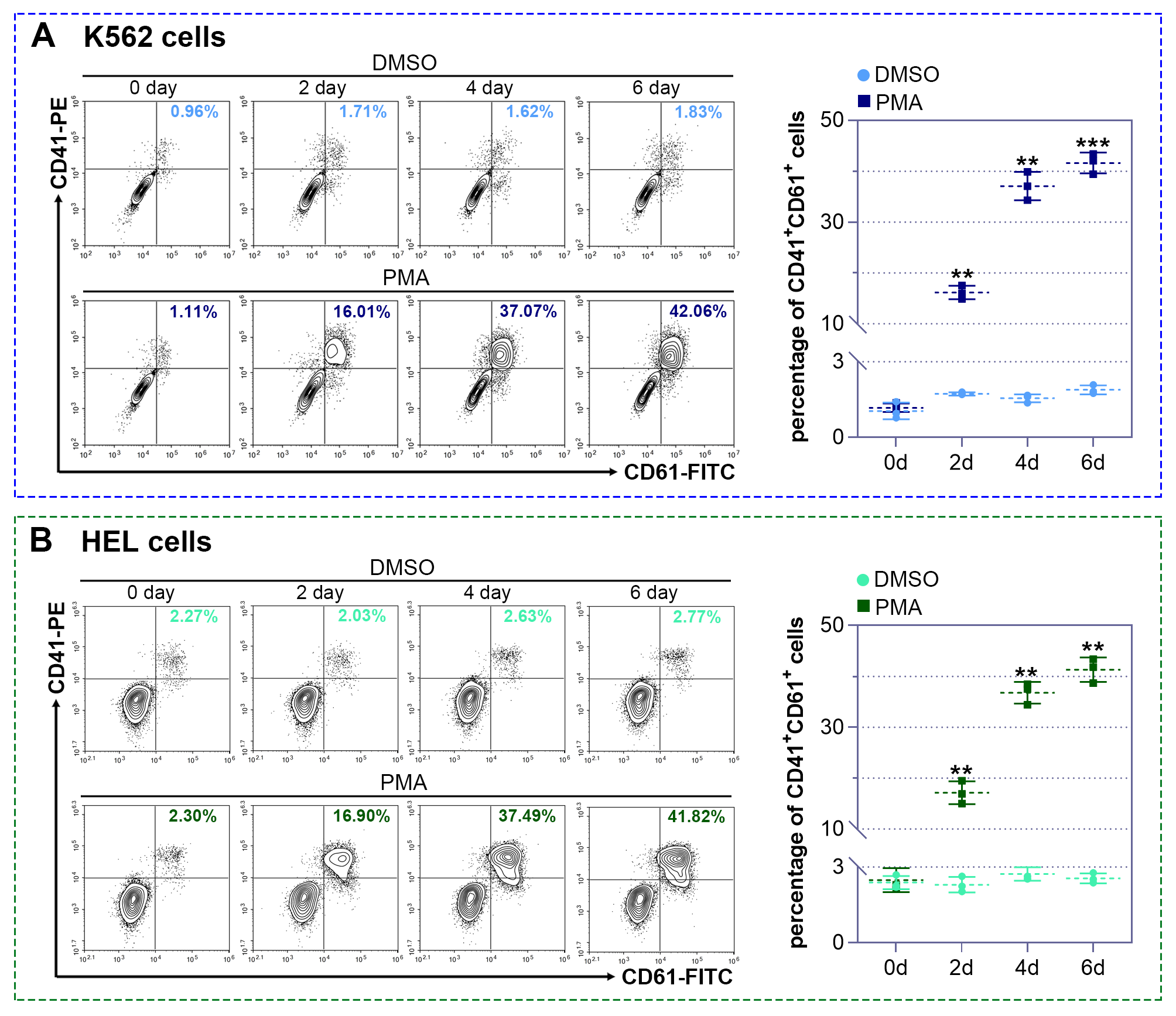

Supplement: Supplementary file 1 — Supplementary Material 1. Sup. Figure-1. Phorbol 12-myristate 13-acetate (PMA) treatment induces the expression of the megakaryocytic markers CD61 and CD41. Representative contour plots were used to identify CD41+/CD61+ cells in two human erythroleukemic cell lines, K562 (A) and HEL (B), after treatment with 25 nM of PMA for 2 days, 4 days, and 6 days. [file 40364_2024_614_MOESM1_ESM.tif]

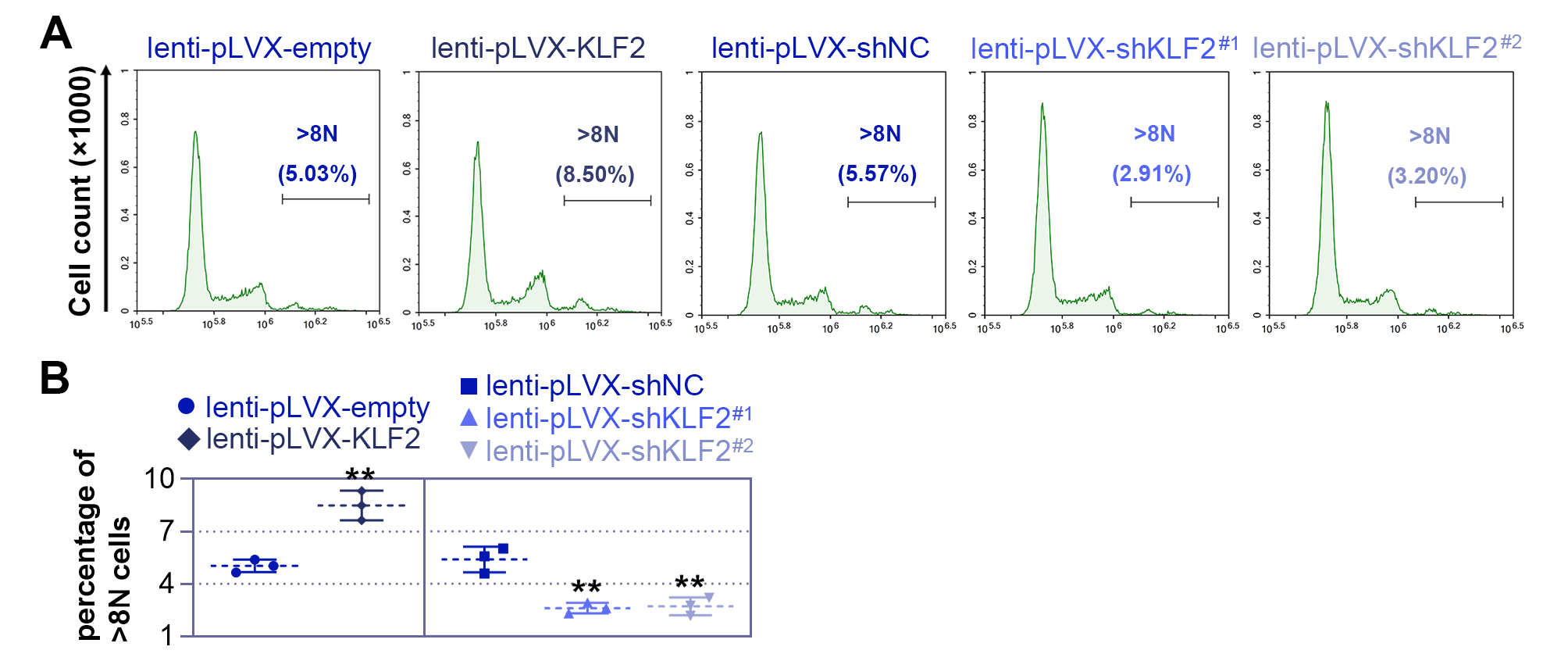

Supplement: Supplementary file 2 — Supplementary Material 2. Sup. Figure-2. Krüppel-like factor 2 (KLF2) regulates DNA ploidy after 6 days of phorbol 12-myristate 13-acetate (PMA) treatment. Lentiviral particles were infected into K562 cells for 72 h, and the cells were treated with 25 nM phorbol 12-myristate 13-acetate (PMA) for another 6 days. Subsequently, DNA ploidy was assessed by flow cytometry (A), and cells with > 8N DNA were counted (B). [file 40364_2024_614_MOESM2_ESM.tif]

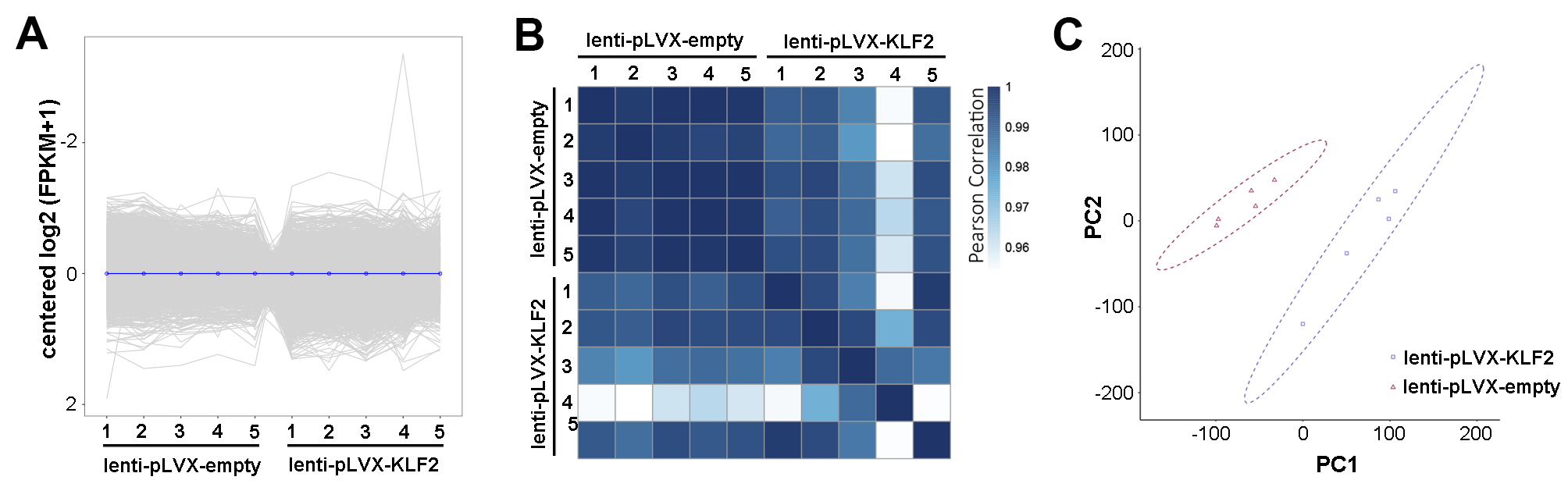

Supplement: Supplementary file 3 — Supplementary Material 3. Sup. Figure-3. krüppel-like factor 2 (KLF2) leads to significant transcriptome changes. K562 cells were infected with lenti-pLVX-empty and lenti-pLVX-KLF2 for 72 h, then treated with 25 nM PMA for 6 days to induce megakaryopoiesis. mRNA-sequencing was performed to explore the changed transcriptome expression patterns induced by KLF2 overexpression. A. Overall expressed patterns. The abscissa represents different samples, and the vertical axis represents the centered log2 (FPKM + 1). B. A heatmap of the correlation in the overall gene expression among samples. C. A principal component analysis (PCA) plot based on the gene expression profile among samples. PC1 represents 41.49% variance, and PC2 represents 15.4% variance. [file 40364_2024_614_MOESM3_ESM.tif]

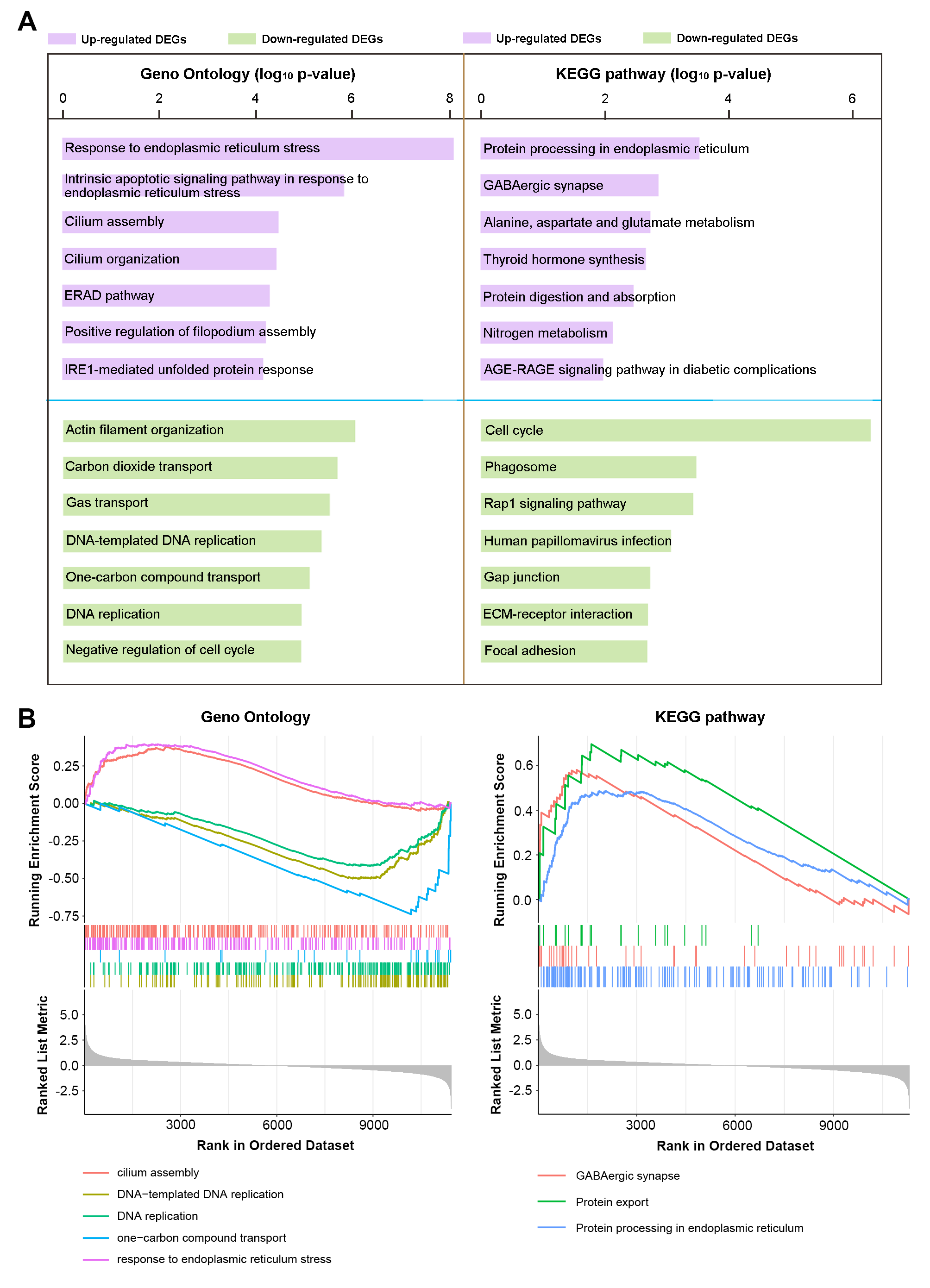

Supplement: Supplementary file 4 — Supplementary Material 4. Sup. Figure-4. Functional enrichment suggests the biological processes and pathways influenced by krüppel-like factor 2 (KLF2). A. Gene Ontology (GO) and Kyoto Encyclopedia of Genes and Genomes (KEGG) enrichment analysis of the up-regulated and down-regulated differentially expressed genes (DEGs). The top 7 enriched pathways are shown in a bar chart. B. Gene Set Enrichment Analysis (GSEA) shows the top 5 enriched GO terms and 3 enriched KEGG pathways. [file 40364_2024_614_MOESM4_ESM.tif]

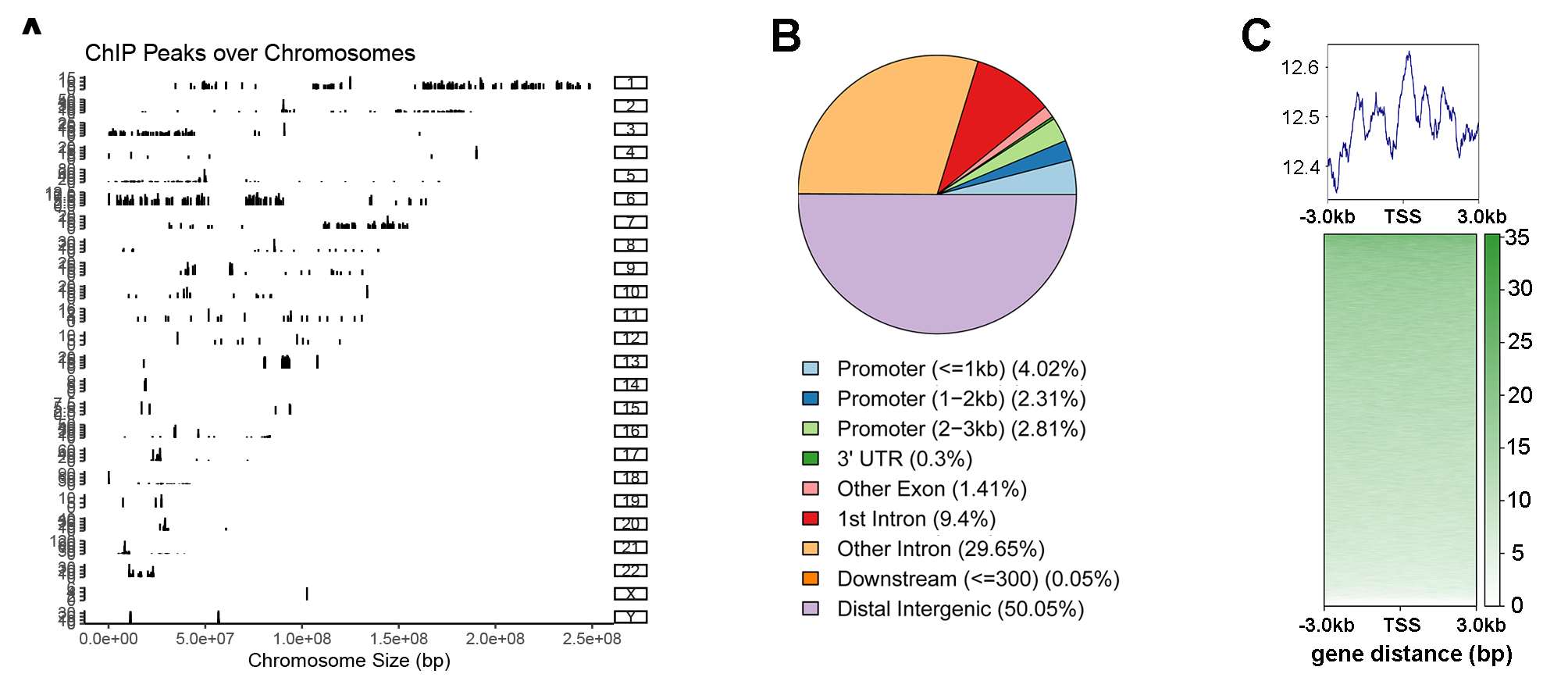

Supplement: Supplementary file 5 — Supplementary Material 5. Sup. Figure-5. Chromatin immunoprecipitation-sequencing (ChIP-seq) suggests genes that bind with krüppel like factor 2 (KLF2). K562 cells were infected with lenti-pLVX-KLF2 for 72 h, then treated with 25 nM PMA for 6 days to induce megakaryopoiesis. ChIP-seq was performed to explore the binding DNAs of KLF2. A. The location of all ChIP peaks over the chromosome. B. Peaks annotated to the expressed genes are categorized into regions according to the distance from their transcription start site (TSS). A pie chart shows the percentage of peaks in each region. C. Heatmap of the KLF2 ChIP-seq signals at gene promoters (-3 kb ~ 3 kb). [file 40364_2024_614_MOESM5_ESM.tif]
